# The Influence of Brand Image and Favorability Toward Citizens in a Product’s Country of Origin on Product Evaluation: Moderating Effects of Switching Costs

**DOI:** 10.3389/fpsyg.2022.740269

**Published:** 2022-03-01

**Authors:** Yan Shen, Riaz Ahmad

**Affiliations:** ^1^School of Economics, Yunnan Minzu University, Kunming, China; ^2^School of Education, Yulin University, Yulin, China

**Keywords:** brand image, country of origin, product evaluation, repurchase intention, switching costs

## Abstract

This study aimed to provide practical implications for South Korean corporations seeking to enter the Chinese market. It explored the influences of brand image and favorability toward citizens in a product’s country of origin (FCPCO) on consumers’ product evaluation and repurchase intention, in addition to examining the moderating effects of procedural switching costs (economic risk costs, evaluation costs, learning costs, and set-up costs), financial switching costs (benefit loss costs and monetary loss costs), and relational switching costs (personal relationship loss costs and brand relationship loss costs) on the aforementioned influences. Although previous studies have established the relationships between some of the aforementioned variables, further research is required to determine the moderating effects of switching costs in various dimensions. Studies on the relationships of a product’s country of origin with product evaluation and repurchase intention have rarely explored FCPCO. Through a questionnaire survey, this study obtained effective data from 302 respondents. Constituted of an exploratory research design, this study adopted PLS-SEM method for empirical analysis. IPMA analysis results indicated that brand image had a stronger influence on product evaluation than FCPCO did and that FCPCO had a stronger influence on repurchase intention than brand image did. Overall, the performance of FCPCO was higher than that of brand image. Moreover, economic risk costs and brand relationship loss costs positively moderated the relationship between brand image and product evaluation; monetary loss costs and brand relationship loss costs negatively moderated the relationship between FCPCO and product evaluation. These study results could help corporations gain competitive edge.

## Introduction

Economic globalization, which epitomizes the advance of human civilization, is an inevitable outcome of economic growth at the global scale. The Regional Comprehensive Economic Partnership, which came into effect on November 15, 2020, is a critical step toward an open global economy and the stabilization of the global economy. With the continual expansion of geographical boundaries in the global economy and the formation and reformation of trade blocs, the association and interdependence between different regions have strengthened substantially. Through the formation of strategic alliances and the acquisition of foreign direct investment, business operations can be upgraded to a global scale, which contributes to the diversification of products in the international market. Investigating the evaluation and repurchase intention of foreign products is crucial in a society facing substantial growth in international commerce. Moreover, a widely held belief is that retaining an old customer is more economic than acquiring a new one (Qiu et al., 2015; Ghazali et al., 2016). Market globalization has aroused many changes in the purchasing environment of consumers, and a variety of product information directly faced by the consumers makes the factors influencing consumers’ purchase intention and product evaluation more complicated. Product price, functionality, and quality are no longer the only considerations in product evaluation. Numerous other factors, including brand image, the image of a product’s country of origin, and switching costs, are considered in product evaluation. In an increasingly active and competitive market, the best core marketing strategy is to provide excellent value that meets the needs of existing clientele for maintaining their loyalty (Chuah et al., 2017). Therefore, marketing research is focused on product evaluation and purchase intention or behavior (Iglesias et al., 2019). Identifying factors that affect product evaluation and repurchase intention is a critical task for researchers and marketing agents because these two aspects influence the profitability and competitive edge of a corporation.

Typically, consumers’ decision-making process for a purchase comprises the following phases: problem introduction, information search, appraisal of alternatives, decision-making, and post purchase behavior (Kotler and Armstrong, 2010). Product evaluation, purchase intention, and purchase behavior are major aspects of marketing theories (Iglesias et al., 2019). Corporations often send certain messages to consumers to form a powerful, unique, and advantageous bond between consumers and their products (Suh and Youjae, 2006). In a global market, although consumers have access to a large amount of information on a variety of products, they have limited time and energy and thus can only rely on a small number of clues that reflect the product quality for making a purchase decision (Ozretic-Dosen et al., 2007). A product’s country of origin and brand image are external clues that are considered to be indicators of product quality; thus, these factors serve as bases for simplifying the information that must be processed in product evaluation (Hsieh et al., 2004; Ozretic-Dosen et al., 2007). Consequently, the aforementioned factors influence consumers’ purchase decisions (Ozretic-Dosen et al., 2007; Song et al., 2019), and the importance of these factors has been widely acknowledged (Roth et al., 2008; Kim et al., 2017; Nghiêm-Phú and Nguyễn, 2020). The theory of consumption values (e.g., functional, affective, social, and cognitive values) can adequately explain consumers’ choice of one product over another (Wong et al., 2019). Information on the brand and country of origin of a product affects consumers’ perceived value of the product, which in turn affects their purchase decision. If the brand or country of origin of a product has a positive image, high customer loyalty is developed toward the product (Yasin et al., 2007; Song et al., 2019). Economic growth has caused products to become increasingly complicated and standardized, which has compelled corporations to emphasize the brand and country of origin of a product in their international marketing strategies to achieve greater market acceptance and thus greater business success (Webb and Po, 2000). The image of a country’s citizens is an element of a country’s national image (Martin and Eroglu, 1993). Because a country is formed by its citizens, the favorable sentiment toward a country’s citizens among consumers can induce consumers to rate the country’s products highly and buy them repeatedly. Limited studies have been conducted on how sentiment toward citizens of a product’s country of origin (FCPCO) affects consumers’ product evaluation and repurchase intention; therefore, the present study attempted to fill this research gap. Moreover, this study compared the influences of FCPCO and brand image on product evaluation and repurchase intention.

When purchasing a new product, consumers consider their experience with existing products, the attractiveness of alternatives, and the cost of replacing existing products (Kim and Lee, 2017). Switching costs have been recognized as a powerful instrument of defensive marketing for establishing a long-term relationship with customers and increasing revenue. Therefore, such costs are a key indicator of customer retention ability (Kim and Lee, 2017; Temerak and El-Manstrly, 2019; Kim et al., 2020). Switching costs also serve to improve corporate competitiveness (Nie et al., 2018). In an increasingly competitive market where consumers have easy access to numerous alternative options (Ghazali et al., 2016). The role of switching costs is particularly critical when several viable options are available to consumers (Lee et al., 2001). A long-term consumer-product relationship provides additional benefits to consumers. For example, under a long-term consumer-product relationship, consumers can use the relevant product with high confidence, have their needs and preferences better met by the product, and expect to receive preferential treatment from the company manufacturing the product (Bell et al., 2005). Under certain circumstances, an antecedent of loyalty does not necessarily lead to loyalty. For example, satisfaction does not necessarily lead to loyalty, and dissatisfaction does not necessarily lead to a change of choice. Therefore, to gauge the variability of the satisfaction-loyalty relationship, the moderating variables of this relationship must be investigated. Switching costs are one variable that moderate the aforementioned relationship (Chuah et al., 2017). Numerous studies have confirmed that switching costs moderate the relationships of loyalty with satisfaction (Chang and Chen, 2008; Qiu et al., 2015; Chuah et al., 2017), perceived value (Yang and Peterson, 2004; El-Manstrly, 2016), and trust (Aydin et al., 2005). Moreover, switching costs moderate the relationships of repurchase intention with satisfaction (Jones et al., 2000; Blut et al., 2015), quality (Wang, 2010), and perceived value (Wang, 2010); the relationship of trust with continuance commitment (Laksamana et al., 2013); the relationship of satisfaction with word-of-mouth intentions (Han and Ryu, 2012); and the relationship of brand equity with purchase intentions (Chen and Chang, 2008). Thus, switching costs act as a quasi-moderating factor of loyalty (Ghazali et al., 2016). A positive brand image and national image can increase consumers’ perceived value of and satisfaction with a product. The available knowledge regarding trust and studies on switching costs indicate that switching costs possibly exert moderating effects on the relationships of brand image and FCPCO with product evaluation and repurchase intention. Most studies on the moderating effects of switching costs have conceptualized switching costs as a one-dimensional construct (Back and Lee, 2009; Wang, 2010; Qiu et al., 2015). By contrast, switching costs should be considered a complicated, multidimensional construct because the results obtained by treating them as a one-dimensional construct have been unsatisfactory (Ghazali et al., 2016). Switching costs affect user behavior differently in different dimensions; however, the effects of subcategories of switching costs have only attracted limited academic attention. Similarly, no study has been conducted on the moderating effects of switching costs on variables related to brand image and a product’s country of origin. To fill this research gap, this study investigated the moderating effects of switching costs in multiple dimensions for verifying if these costs moderate the relationships of brand image and FCPCO with product evaluation and repurchase intention. The findings of this study are expected to help suppliers in South Korea gain a further understanding of the relationship-building process with their Chinese customers (Huifeng and Ha, 2020) and increase their profits through the control of switching costs.

This study adopted the classification system proposed by Burnham et al. (2003) to divide switching costs into procedural switching costs (economic risk costs, evaluation costs, learning costs, and set-up costs), financial switching costs (benefit loss costs and monetary loss costs), and relational switching costs (personal relationship loss costs and brand relationship loss costs) for examining their moderating effects on the relationships of brand image and FCPCO with product evaluation and repurchase intention. The current study also explored how brand image and FCPCO affect product evaluation and repurchase intention.

## Literature Review and Hypothesis Development

### Relationships of Brand Image With Product Evaluation and Repurchase Intention

Brand image can be regarded as the image created in consumers’ minds by brand associations (Hsieh et al., 2004), or it can be regarded as a brand’s overall impression on consumers (Nisar and Whitehead, 2016). Brand image influences consumers’ product choices (Padgett and Allen, 1997; Cretu and Brodie, 2007). A trustworthy information label often influences consumers’ attitude toward and purchase decision regarding a product (Buda and Zhang, 2000). Studies have extensively verified the importance of brand name (or image) in consumers’ product evaluation. Consumers that do not have a specific preference for a product usually rely on brand name to infer product quality (Jacoby et al., 1971; Szybillo and Jacoby, 1974). Individuals with in-depth knowledge on brands tend to rely considerably more on brand names to judge product quality than do individuals who know little about brands (Grewal et al., 1998). Brand reputation effectively conveys messages on product or service quality to a target audience (Kwun and Oh, 2007). Thus, brand reputation significantly and positively influences product evaluation (Estes et al., 2018). Consistency between brand image and consumers’ self-image positively influences product evaluation (Graeff, 1996). Brand loyalty represents strong evidence regarding the importance of brand name in consumers’ product evaluation (Lee and Ganesh, 1999). The aforementioned discussion indicates that brand image has a positive effect on product evaluation.

Repurchase intention refers to customers’ intention of purchasing a product or service that they have previously used. It can be regarded as customers’ psychological commitment to a product or service (Erkan and Evans, 2016). In the field of brand marketing, consumers’ repurchase intention has been extensively studied because it affects corporate profitability. Studies have noted that brand image positively and directly affects repurchase intention (Huang et al., 2019) and that brand image directly and indirectly affects customer loyalty (Johnson et al., 2000; Cretu and Brodie, 2007; Da Silva and Alwi, 2008). Accordingly, the following hypotheses are proposed in this study:

*H1:* Brand image has a positive effect on product evaluation.

*H2:* Brand image has a positive effect on repurchase intention.

### Relationships of FCPCO With Product Evaluation and Repurchase Intention

Increased international commerce has resulted in the exposure of numerous consumers to foreign goods with unfamiliar origin (Escandon-Barbosa and Rialp-Criado, 2019). Because most of such goods originate from an environment that consumers cannot relate to, consumers tend to evaluate these goods according to the image of the country of origin of the goods (Etkin and Sela, 2016). Schooler (1965) was the first to emphasize the importance of a product’s country of origin in consumers’ product selection. Country image, which is broad and all-inclusive, provides consumers clues about a product’s country of origin (Roth and Romeo, 1992). A positive country image enhances the status of a product and reduces the perceived risks associated with its purchase (Ahmed et al., 2002; Elango and Sethi, 2007). Studies on the image of a product’s country of origin can be divided into three categories: studies focused on a cognitive, affective, or conative component of national image. These components serve as nodes in consumers’ memories that connect and interact with other nodes. Cognitive components of country image encompass consumers’ perceptions on the industrial development and technological advancement of a country; affective components of country image encompass consumers’ affective response to the citizens of a country; and conative components of country image encompass consumers’ anticipated level of interaction with the citizens of a country (Laroche et al., 2005; Escandon-Barbosa and Rialp-Criado, 2019). FCPCO is an affective component of national image. Although the effects of national image on consumers’ product evaluation and repurchase intention have been studied extensively, studies have not examined the effects of FCPCO on consumers’ product evaluation and repurchase intention.

Information on a product’s country of origin has a considerable influence on consumers’ product evaluation, preference, selection, and purchase intention (Yasin et al., 2007). The country of origin serves as a clue for product evaluation. It forms the basis of consumers’ perception of the overall quality of products, and consumers evaluate a product largely on the basis of this perception (Ahmed et al., 2002; Usunier, 2006). Demographic characteristics influence the effect of the country of origin on product evaluation in three ways: women tend to rate foreign products higher than men do (Mittal and Tsiros, 1995); individuals with a high educational level are more inclined to evaluate foreign products than are those with a low education level (Mittal and Tsiros, 1995); and individuals with a high income more actively evaluate foreign products than do those with a low income (Han and Terpstra, 1988). Moreover, characteristics, such as consumers’ product familiarity and psychological characteristics, may affect product evaluation (Peterson and Jolibert, 1976). For example, consumers’ product evaluation can be affected by FCPCO. In this study, the favorability of an individual was regarded as the attractiveness of an object perceived by the individual. Therefore, the concept of interpersonal attraction was referenced to define favorability as an individual’s tendency to actively or passively rate another individual or what an individual represents (Walster et al., 1978); thus, favorability represents an individual’s active or passive attitude toward another individual (Islam et al., 2019). Accordingly, FCPCO represents an individual’s active or passive attitude toward the citizens of other country. The effect of country of origin has been widely exploited by corporations to sway consumers’ purchase intention (Lee and Robb, 2019). Numerous studies have confirmed that the image of a product’s country of origin can affect consumers’ evaluations of and opinions on the product (Essoussi and Merunka, 2007; Escandon-Barbosa and Rialp-Criado, 2019), which in turn affect their purchase intention (Bilkey and Nes, 1982; Roth and Romeo, 1992) and loyalty (Yasin et al., 2007; Lee et al., 2011). FCPCO constitutes the information on a product’s country of origin and thus can affect consumers’ repurchase intention to a certain degree. From another viewpoint, if consumers have a positive impression of the citizens of a country, their trust in products from that country increases, which contributes to increased product loyalty (Agustin and Singh, 2005; Han and Hyun, 2013). Accordingly, this study proposes the following hypotheses:

*H3:* FCPCO has a positive effect on product evaluation.

*H4:* FCPCO has a positive effect on repurchase intention.

### Moderating Effects of Switching Costs

The concept of switching costs was first proposed by Jackson (1985), who stated that switching costs represent an increase in the possibility of successful customer retention. Switching costs are the nonrecurring costs that consumers incur when switching from one supplier to another (Burnham et al., 2003). Numerous methods can be used to classify switching costs. The mostly widely used and comprehensive method for classifying switching costs is the method proposed by Burnham et al. (2003). Therefore, this method was adopted in the current study to divide switching costs into procedural switching costs, financial switching costs, and relational switching costs.

Procedural switching costs refer to switching costs that require the investment of time and efforts. Four types of procedural switching costs exist as, economic risk costs, evaluation costs, learning costs, and set-up costs (Burnham et al., 2003). Specifically, economic risk costs are the costs of negative outcomes resulting from the uncertainties caused by a lack of information (Jackson and Bund, 1985; Klemperer, 1995); evaluation costs are the time and efforts required for research and exploration after consumers decide to change a supplier (Burnham et al., 2003); learning costs are the time and efforts required to familiarize oneself with a new product (Burnham et al., 2003); and set-up costs refer to the costs incurred by consumers in beginning a relationship with a new supplier or the time and efforts required by consumers to start using a new product (Guiltinan, 1989; Klemperer, 1995).

Financial switching costs, which comprise benefit loss costs and monetary loss costs, are quantified losses in financial resources (Burnham et al., 2003). Benefit loss costs refer to the economic benefits that consumers can continue to enjoy if they retain their original supplier. Thus, benefit loss costs are related to consumers’ contract with the original supplier (Guiltinan, 1989). For example, consumers who switch to a new supplier would lose the credits that they have accumulated and the preferential treatment for old customers (Guiltinan, 1989). Monetary loss costs refer to the one-off monetary expenses associated with switching to a new supplier, such as the starting fees and deposits that must be paid to a new supplier (Jackson and Bund, 1985; Guiltinan, 1989).

Relational switching costs refer to the mental or psychological readjustment required to adapt to the loss of a relationship or identity (Burnham et al., 2003). Therefore, they comprise personal relationship loss costs and brand relationship loss costs. Personal relationship loss costs are the affective or emotional losses associated with the breaking of an established tie between consumers and their original supplier (or rather, the supplier’s staff that serve them; Guiltinan, 1989; Klemperer, 1995). Brand relationship loss costs refer to consumers’ affective or emotional losses associated with the breaking of an established tie (identity) with a brand (Aaker, 1992; Burnham et al., 2003).

Researchers have not reached a consensus on the moderating effects of switching costs. Some researchers have reported that switching costs exert a positive moderating effect on the relationship between satisfaction and loyalty (Lee et al., 2001; Yang and Peterson, 2004; Chou and Lu, 2009; de Matos et al., 2013), whereas others have reported the switching costs exert a negative moderating effect on the aforementioned relationship (Dagger and David, 2012; Chuah et al., 2017). Moreover, some studies have reported that switching costs (learning costs, artificial costs, uncertainty costs, search and evaluation costs, and brand relationship loss costs) do not moderate the relationship between satisfaction and loyalty (Ghazali et al., 2016), whereas some other studies have reported that switching costs moderate the relationship between satisfaction and loyalty only under certain conditions (Yang and Peterson, 2004). Shi et al. (2015) investigated the moderating effects of switching costs subdivided at multiple dimensions, indicating that economic risk costs, evaluation costs, learning costs, set-up costs, benefit loss costs, monetary loss costs, personal relationship loss costs, brand relationship loss costs, and social ties loss costs can moderate the relationship between satisfaction and customer loyalty. Studies on the moderating effect of switching costs on the relationship between perceived value and loyalty have also obtained inconsistent findings. One study found that switching costs exert a nonsignificant moderating effect on the relationship between perceived value and member loyalty (Back and Lee, 2009), whereas another study found that switching costs exert a negative moderating effect on the aforementioned relationship (Stan, 2015). In addition, the study of Machado and Pinheiro (2013) revealed that pre-switching search and evaluation costs enhance the influences of perceived quality and professional services on customer loyalty. Moreover, studies on the moderating effect of switching costs in different dimensions have obtained inconsistent results. Blut et al. (2015) reported that procedural switching costs and relational switching costs negatively moderate the relationships of satisfaction with repurchase intentions and repurchase behavior, whereas financial switching costs exert a positive moderating effect on the aforementioned relationships. El-Manstrly (2016) reported that financial switching costs positively moderate the relationships of customer loyalty with trust and customers’ perceived value (for specific services); that procedural switching costs have a positive or negative mediating effect on the relationship between customers’ perceived value and customer loyalty for certain service types; and that relational switching costs do not exert a moderating effect. Vasudevan et al. (2006) noted that relational switching costs weaken the effect of satisfaction on commitment. Chen and Chang (2008) discovered that switching costs moderate the relationship between brand equity and purchase intentions. A positive brand image and high FCPCO enhance consumers’ satisfaction with and perceived value of the products of a country. Thus, switching costs may exert moderating effects on the relationships of brand image and FCPCO with repurchase intention. Furthermore, studies on the moderating effect of switching costs on word-of-mouth indicate that switching costs positively moderate the relationship between user attachment to travel apps and word-of-mouth (Zhang et al., 2019) and switching costs (monetary switching costs and nonmonetary switching costs) negatively moderate the relationships between service encounter performance and word-of-mouth intentions and between satisfaction and word-of-mouth intentions (Han and Ryu, 2012). Thus, switching costs exert moderating effects on the relationships of brand image and FCPCO with product evaluation. Accordingly, this study proposes the following hypotheses:

*H5:* Switching costs moderate the relationship between brand image and product evaluation.

*H5-1:* Procedural switching costs (economic risk costs, evaluation costs, learning costs, and set-up costs) moderate the relationship between brand image and product evaluation.

*H5-2:* Financial switching costs (benefit loss costs and monetary loss costs) moderate the relationship between brand image and product evaluation.

*H5-3:* Relational switching costs (personal relationship loss costs and brand relationship loss costs) moderate the relationship between brand image and product evaluation.

*H6:* Switching costs moderate the relationship between brand image and repurchase intention.

*H6-1:* Procedural switching costs (economic risk costs, evaluation costs, learning costs, and set-up costs) moderate the relationship between brand image and repurchase intention.

*H6-2:* Financial switching costs (benefit loss costs and monetary loss costs) moderate the relationship between brand image and repurchase intention.

*H6-3:* Relational switching costs (personal relationship loss costs and brand relationship loss costs) moderate the relationship between brand image and repurchase intention.

*H7:* Switching costs moderate the relationship between FCPCO and product evaluation.

*H7-1:* Procedural switching costs (economic risk costs, evaluation costs, learning costs, and set-up costs) moderate the relationship between FCPCO and product evaluation.

*H7-2:* Financial switching costs (benefit loss costs and monetary loss costs) moderate the relationship between FCPCO and product evaluation.

*H7-3:* Relational switching costs (personal relationship loss costs and brand relationship loss costs) moderate the relationship between FCPCO and product evaluation.

*H8:* Switching costs moderate the relationship between FCPCO and repurchase intention.

*H8-1:* Procedural switching costs (economic risk costs, evaluation costs, learning costs, and set-up costs) moderate the relationship between FCPCO and repurchase intention.

*H8-2:* Financial switching costs (benefit loss costs and monetary loss costs) moderate the relationship between FCPCO and repurchase intention.

**Figure 1:**
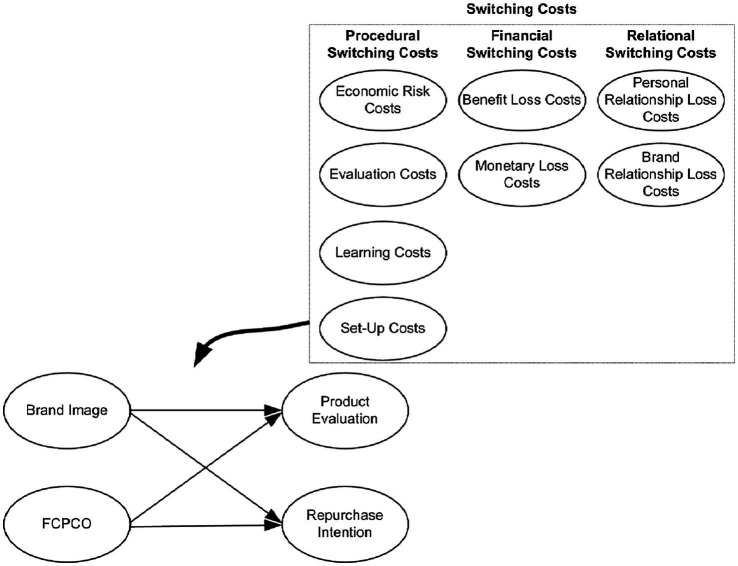
Researcher model.

*H8-3:* Relational switching costs (personal relationship loss costs and brand relationship loss costs) moderate the relationship between FCPCO and repurchase intention Figure 1.

## Research Method

A questionnaire was designed based on the above research hypotheses, with 20 participants with experience in using Korean products and services invited to complete the prediction. Then, ambiguous contents and those that might be subject to misunderstanding were modified, enabling participants to fully understand the content and improving the quality of the questionnaire. After completing the first draft of the questionnaire, a pilot test was carried out at a university in Beijing to ensure that participants would not misunderstand the questionnaire, in which 100 valid samples were collected. Based on the reliability coefficient criteria recommended by Nunnally and Bernstein (1978), the Cronbach’s alpha value of the pilot rest exceeded 0.7, implying acceptable internal consistency and stability of the questionnaire. As network connections in some areas were unstable, the questionnaire was distributed online and offline to Chinese consumers with experience in using Korean products and services (such as Korean cosmetics and plastic surgery hospitals). Back translation was conducted to ensure the accuracy of the questionnaire items (i.e., the items were translated from English into Chinese and then translated back into English; Brislin, 1986). The focus of this study was the effect of Korean brands at the national level, so all products and services from South Korea were available, not confined to a specific brand. For instance, all Japanese products are of high quality. All participants were rewarded with a USB flash drive or a small fortune (*via* telegraphic transfer) to increase their willingness and credibility. Besides, repeated filling in of the questionnaire by the same participant was prevented through filtration of repeat emails. In addition, invalid questionnaires obviously filled in at random or with many missing values were eliminated to improve the quality of survey data. As to content validity, a research model was designed in this study based on relevant dimensions used in previous literatures. The questions in the questionnaire were developed based on literatures and pre-validated scales. Moreover, the questionnaire was carefully verified and modified by many experts in the field of marketing, so that the content validity of the questionnaire was verified appropriately. The questionnaire was administered between September 13 and October 18, 2020. A total of 328 copies of the questionnaire were distributed, and 302 copies valid questionnaires were retrieved. Table 1 presents the questionnaire items. Frequency analysis was conducted using SPSS 20.0. Table 2 presents the demographic characteristics of the respondents.

**Table 1:** Questionnaire measurement items.

Construct	Measurement	References
Brand Image (BI)	BI1. Korean brands are fashionable and popular. BI2. Korean brand quality has favorable reputation. BI3. Korean brands are elegant. BI4. Korean brands are sophisticated.	Low and lamb 2000 Cretu et al. 2007
Favorability toward Citizens in a Product’s Country of Origin (FCPCO)	FCPCO1. What is your impression of Koreans? FCPCO2. What happens if you encounter a Korean on the street? FCPCO3. How do you feel about frequent contact with Koreans? FCPCO4. What would you do if you were required to solve a difficult problem with a Korean? FCPCO5. What do you think of Koreans as friends who study together with you? FCPCO6. How would you feel about adding Koreans to your circle of friends? FCPCO7. How would you feel about having Korean as your closest friends? FCPCO8. How would you feel about having a Korean as your confidant?	McCroskey and McCain 1974 Berscheid and Hatfield 1969 Hwang 1996
Product Evaluation (PE)	PE1. The quality of the Korean products (or services) I use is satisfactory. PE2. The Korean products (or services) I use have excellent durability. PE3. The Korean products (or services) I use are cost-effective.	White 1979 Narayana 1981
Economic Risk Costs (ERC)	ERC1. Replacing Korean products (or services) with products (or services) from other countries may not work well. ERC2. Replacing Korean products (or services) with products (or services) from other countries may result in a poor experience. ERC3. Replacing Korean products (or services) with products (or services) from other countries may incur additional costs. ERC4. Replacing Korean products (or services) with products (or services) from other countries may result in financial losses. ERC5. Replacing Korean products (or services) with products (or services) from other countries may cause unimaginable inconvenience. ERC6. Replacing Korean products (or services) with products (or services) from other countries may have unpredictable final outcomes.	Burnham et al. 2003
Evaluation Costs (EC)	EC1 There is insufficient time to evaluate the information of the alternative products (or services). EC2. It takes considerable time/effort to obtain information to evaluate new products (or services) that replace Korean products (or services). EC3. It takes considerable time/effort to compare the advantages of Korean products (or services) and those of other countries. EC4. It is difficult to compare products (or services) of other countries that replace Korean products (or services).	
Learning Costs (LC)	LC1. It takes considerable time to learn about the functions provided by Korean products (or services) and products (or services) from other countries. LC2. Understanding a new product (or service) does not require much effort. LC3. More effort is needed after changing to using products (or services) from other countries.	
Set-Up Costs (SUC)	SUC1. It takes considerable time to replace Korean products (or services) with products (or services) from other countries. SUC2. Replacing Korean products (or services) with products (or services) from other countries involves an unpleasant sales process. SCU3. Many processes are required to replace Korean products (or services) with new products (or services) from other countries.	
Benefit Loss Costs (BLC)	BLC1. Replacing Korean products (or services) with products (or services) from other countries may result in the loss of various accumulated resources (e.g., points, credits, and services). BLC2. Replacing Korean products (or services) with products (or services) from other countries may result in the loss of various resources (e.g., points, credits, and services) that I have paid for. BLC3. I will lose regular customer discounts if I stop using current Korean products (or services).	
Monetary Loss Costs (MLC)	MLC1. Replacing Korean products (or services) with products (or services) from other countries may incur other initial costs (e.g., set-up fees, handling fees, membership fees, and deposits). MLC2. Replacing Korean products (or services) with products (or services) from other countries requires the payment of additional fees.	
Personal Relationship Loss Costs (PRLC)	PRLC1. Replacing Korean products (or services) with products (or services) from other countries will make me miss the staff that used to serve me. PRLC2. Staff that provide Korean products (or services) are more efficient than staff providing products (or services) from other countries. PRLC3. Current Korean product (or service) providers are crucial to me. PRLC4. I have a great time communicating with staff who provide Korean products (or services).	
Brand Relationship Loss Costs (BRLC)	BRLC1. I like the public image of the Korean product (or service) providers that I currently use. BRLC2. I support companies that provide Korean products (or services).	
Repurchase Intention (RI)	RI1. I have high repurchase intention for the Korean products (or services) that I currently use. RI2. I will repurchase the Korean products (or services) that I currently use. RI3. I am willing to recommend the Korean products (or services) that I currently use to others. RI4. I will continue to purchase them even if the prices of the Korean products (or services) I currently use increase.	Kim et al. 2012 Liu et al. 2005 Fang et al. 2014 Zeithaml et al. 1996

**Table 2:** Sample demographics.

Characteristic	Options	Frequency	Percentage
Gender	Male	129	42.7
	Female	173	57.3
Marital status	Not married	72	23.8
	Married	176	58.3
	Divorce	48	15.9
	Bereavement	6	2.0
Age	Below 20 years old	21	7.0
	20– 29 years old	72	23.8
	30–39 years old	67	22.2
	40–49 years old	99	32.8
	50–59 years old	39	12.9
	60 years old or above	4	1.3

## Data Analysis

In this study, the partial least squares (PLS) method was used to test the stability of the developed model and verify the proposed hypotheses. Analyses were conducted with the PLS algorithm and bootstrapping method by using the SmartPLS 3.0 software package. The PLS algorithm was used in this study for three reasons. First, this study did not have sufficient theoretical support. Second, the use of the PLS algorithm was due to the lack of a classic theoretical model in this study, which was more oriented toward a theoretical exploration. Third, the developed model contained a large number of constructs (Hair et al., 2012).

### Outer Model

As presented in Table 3, all the measurement items had a factor loading of ≥0.5; thus, all the measurement items were retained (Hair et al., 2010). Moreover, the composite reliability and Cronbach’s alpha were greater than 0.7. Therefore, the developed model passed the reliability test (Nunnally and Bernstein, 1994).

**Table 3:** Reliability analysis and convergent validity.

Construct	Measurement items	Factor loading	Cronbach’s alpha	Composite reliability	AVE	Values of *p*
Brand Image (BI)	B1	0.910	0.951	0.964	0.871	0.000
	B2	0.952				0.000
	B3	0.939				0.000
	B4	0.933				0.000
Favorability toward Citizens in a Product’s Country of Origin (FCPCO)	FCPCO1	0.859	0.939	0.950	0.702	0.000
	FCPCO2	0.829				0.000
	FCPCO3	0.783				0.000
	FCPCO4	0.840				0.000
	FCPCO5	0.875				0.000
	FCPCO6	0.847				0.000
	FCPCO7	0.836				0.000
	FCPCO8	0.833				0.000
Product Evaluation (PE)	PE1	0.921	0.924	0.952	0.868	0.000
	PE2	0.953				0.000
	PE3	0.920				0.000
Repurchase Intention (RI)	RI1	0.892	0.902	0.932	0.776	0.000
	RI2	0.938				0.000
	RI3	0.928				0.000
	RI4	0.753				0.000

The convergent validity and discriminant validity were used to test the construct validity. Fornell and Larcker (1981) suggested that the convergent validity must meet the following criteria: (1) factor loadings >0.5 or 0.6, (2) composite reliability or Cronbach’s alpha >0.7, and (3) average variance extracted (AVE) >0.5. The results presented in Table 3 indicate that the constructs of brand image, FCPCO, product evaluation, and repurchase intention met the criteria of Fornell et al. (1981); thus, the developed model had convergent validity. One condition for discriminant validity is that the square root of the AVE must be greater than the interconstruct correlation coefficient (Fornell and Larcker, 1981). As presented in Table 4, the minimum square root of the AVE was 0.881, which is greater than the interconstruct maximum correlation coefficient of 0.795; thus, the developed model had discriminant validity.

**Table 4:** Correlation of constructs and average variance extracted.

	BI	FCPCO	PE	RI
BI	**0.933**			
FCPCO	0.715	**0.838**		
PE	0.795	0.712	**0.932**	
RI	0.707	0.674	0.693	**0.881**

*BI, brand image; FCPCO, favorability toward citizens in a product’s country of origin; PE, product evaluation; and RI, repurchase intention*; *The diagonal line of the correlation matrix represents the square root of AVE*.

The results of Harman’s single factor analysis indicated that the first factor explained 48.89% of the total variance (i.e., less than 50%; Ylitalo, 2009). The analytical results obtained using SmartPLS 3.0 indicated that all the variance inflation factors between variables were 2.045 (i.e., less than 3.3; Kock and Lynn, 2012). These results suggest that the influence of common method variance was negligible in this study.

### Inner Model

**Figure 2:**
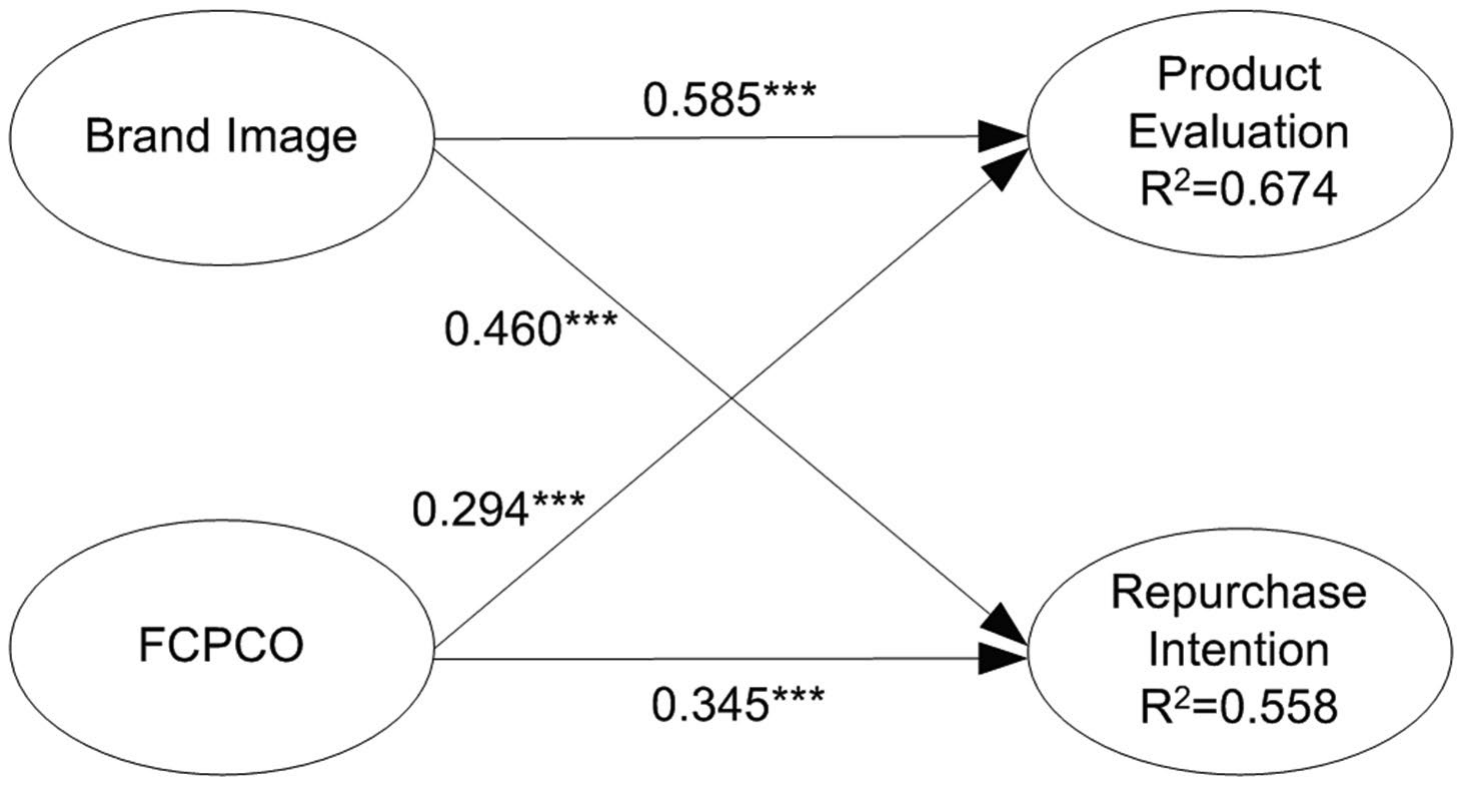
Inner model results (without moderating variables). Solid arrow is significant path; dotted arrow is nonsignificant path. ^*^p < 0.05; ^**^p < 0.01; and ^***^p < 0.001.

Table 5 and Figure 2 present the path coefficient results, including the significance of the path coefficients and the $R^{2}$ values. Brand image positively and significantly affected product evaluation (BI - > PE: β = 0.585, *t* = 11.041, *p* < 0.001); thus, H1 was supported. Brand image positively and significantly affected repurchase intention (BI - > RI: β = 0.460, *t* = 5.462, *p* < 0.001); thus, H2 was supported. FCPCO positively and significantly affected product evaluation (FCPPO - > PE: β = 0.294, *t* = 4.941, *p* < 0.001); thus, H3 was supported. FCPCO positively and significantly affected repurchase intention (FCPCO - > RI: β = 0.345, *t* = 4.734, *p* < 0.001); thus, H4 was supported. The $R^{2}$ values of product evaluation and repurchase intention were 0.674 and 0.558, respectively. For an endogenous latent variable, an $R^{2}$ value of >0.67 indicates practical implications, an $R^{2}$ value of 0.333 represents a moderate explanatory power, and an $R^{2}$ value of 0.19 indicates a weak explanatory power (Urbach and Ahlemann, 2010). Thus, brand image and FCPCO exhibited a moderate to high explanatory power for product evaluation and repurchase intention. Brand image had an $f^{2}$ value of 0.513 for product evaluation and 0.234 for repurchase intention. FCPCO had an $\;f^{2}$ value of 0.130 for product evaluation and 0.132 for repurchase intention. The benchmarks $f^{2}$ values for small, medium, and large substantive impact are 0.020, 0.150, and 0.350, respectively (Urbach and Ahlemann, 2010). Thus, brand image had a large and medium substantive impact on product evaluation and repurchase intention, respectively. By contrast, FCPCO only had a small substantive impact on product evaluation and repurchase intention. The $Q^{2}$ values of product evaluation and repurchase intention were 0.549 and 0.403, respectively. A $Q^{2}$ value of >0 indicates that a model has predictive relevance, whereas a $Q^{2}$ value of <0 indicates that a model lacks predictive relevance (Urbach and Ahlemann, 2010). Product evaluation and repurchase intention exhibited $Q^{2}$ values of >0, which indicated that the developed model had excellent predictive ability. This study used the GoF to test the model fit of the PLS paths (Tenenhaus et al., 2005). The baseline values for the GoF are as follows: ${\mathrm{GoF}}_{small}$ = 0.1, ${\mathrm{GoF}}_{medium}\;$ = 0.25, and ${\mathrm{GoF}}_{large}$ = 0.36 (Wetzels et al., 2009). A GoF value of 0.704 (i.e., >0.36) was obtained, indicating a satisfactory model fit.


$$\mathrm{GOF}=\sqrt{\bar{AVE}\times \bar{R^{2}}}=\sqrt{0.804\times 0.616}=0.704>0.36$$

**Table 5:** Summary of hypotheses testing results.

Hypothesis	Path	Standardized path coefficient	*T* value	Supported
H1	BI - > PE	0.585^***^	11.041	Yes
H2	BI - > RI	0.460^***^	5.462	Yes
H3	FCPCO- > PE	0.294^***^	4.941	Yes
H4	FCPCO- > RI	0.345^***^	4.734	Yes

*BI, brand image; FCPCO, favorability toward citizens in a product’s country of origin; PE, product evaluation; and RI, repurchase intention;* value of ^*^p < 0.05; value of ^**^p < 0.01; and value of ^***^p < 0.001.

**Figure 3:**
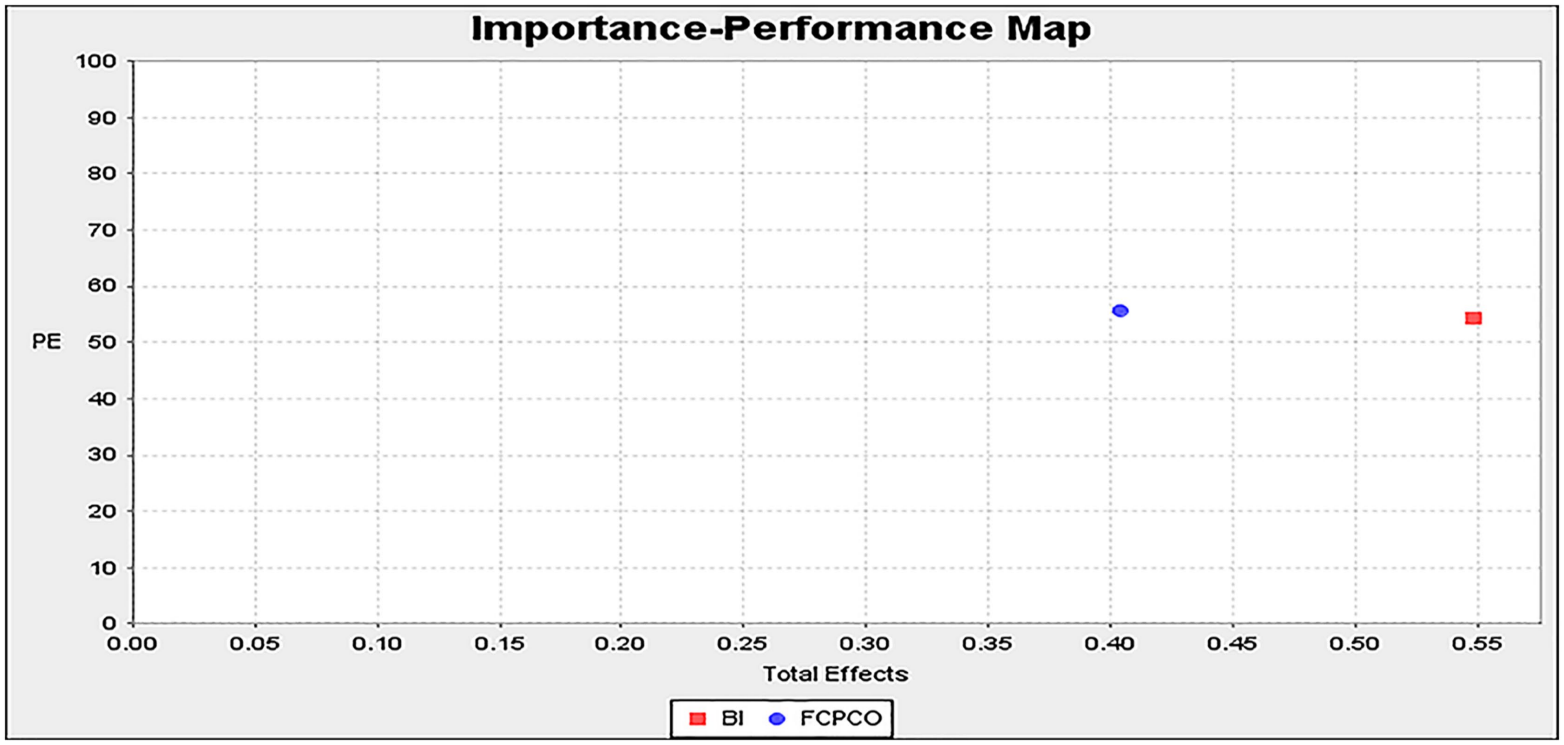
Importance-performance map (product evaluation).

**Figure 4:**
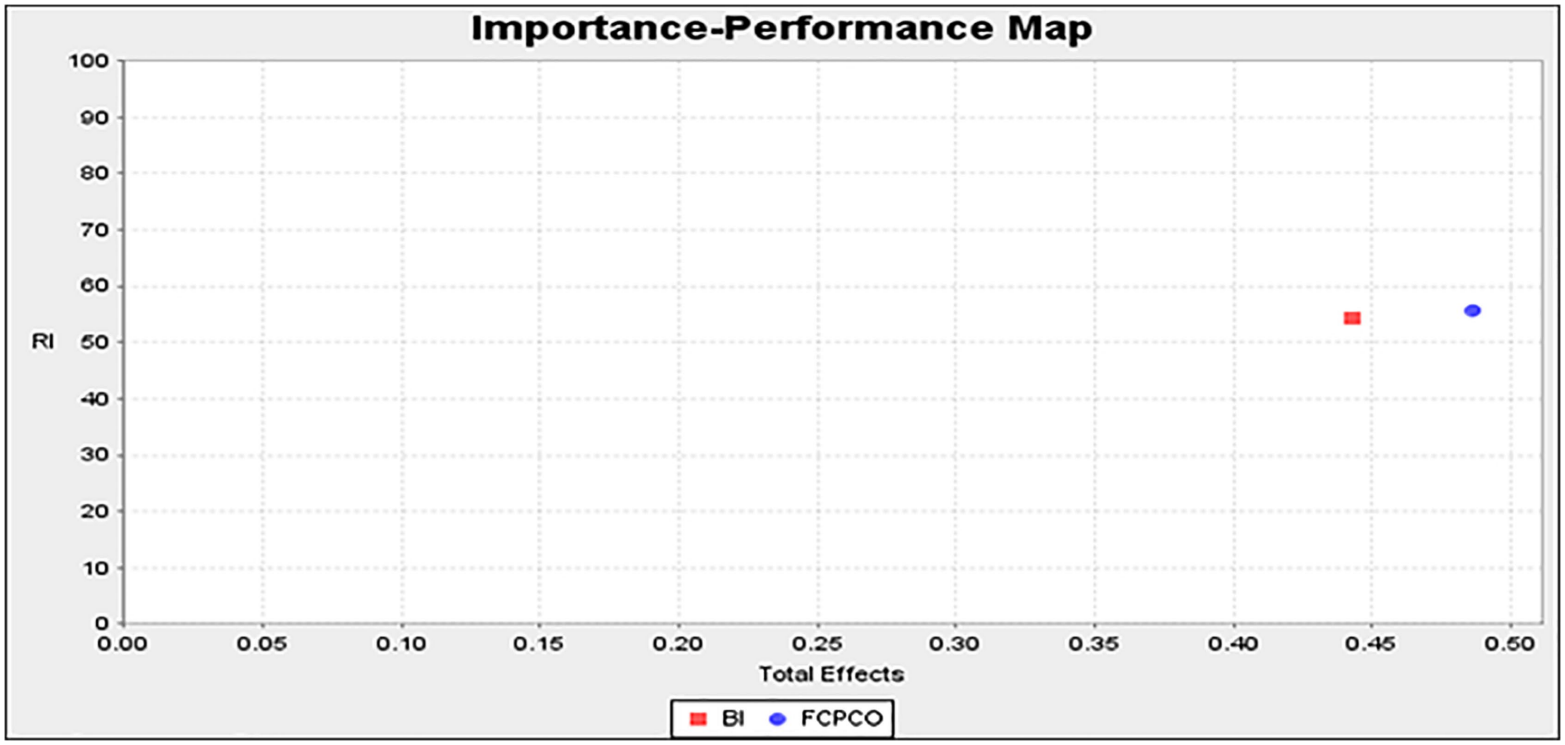
Importance-performance map (repurchase intention).

Figures 3, 4 display the IPMA results, which uses unstandardized results (Ringle and Sarstedt, 2016). For product evaluation, brand image had a performance value of 54.350 and an importance value of 0.548, whereas FCPCO had a performance value of 55.800 and an importance value of 0.403 (Figure 3). Therefore, in terms of product evaluation, brand image had a higher importance value but lower performance value than FCPCO did. For repurchase intention, brand image had a performance value of 54.350 and an importance value of 0.443, whereas FCPCO had a performance value of 55.800 and an importance value of 0.487 (Figure 4). Therefore, in terms of repurchase intention, FCPCO had higher performance and importance values than brand image did. Overall, FCPCO had higher performance values than brand image did.

### Testing of Moderating Effects

**Figure 5:**
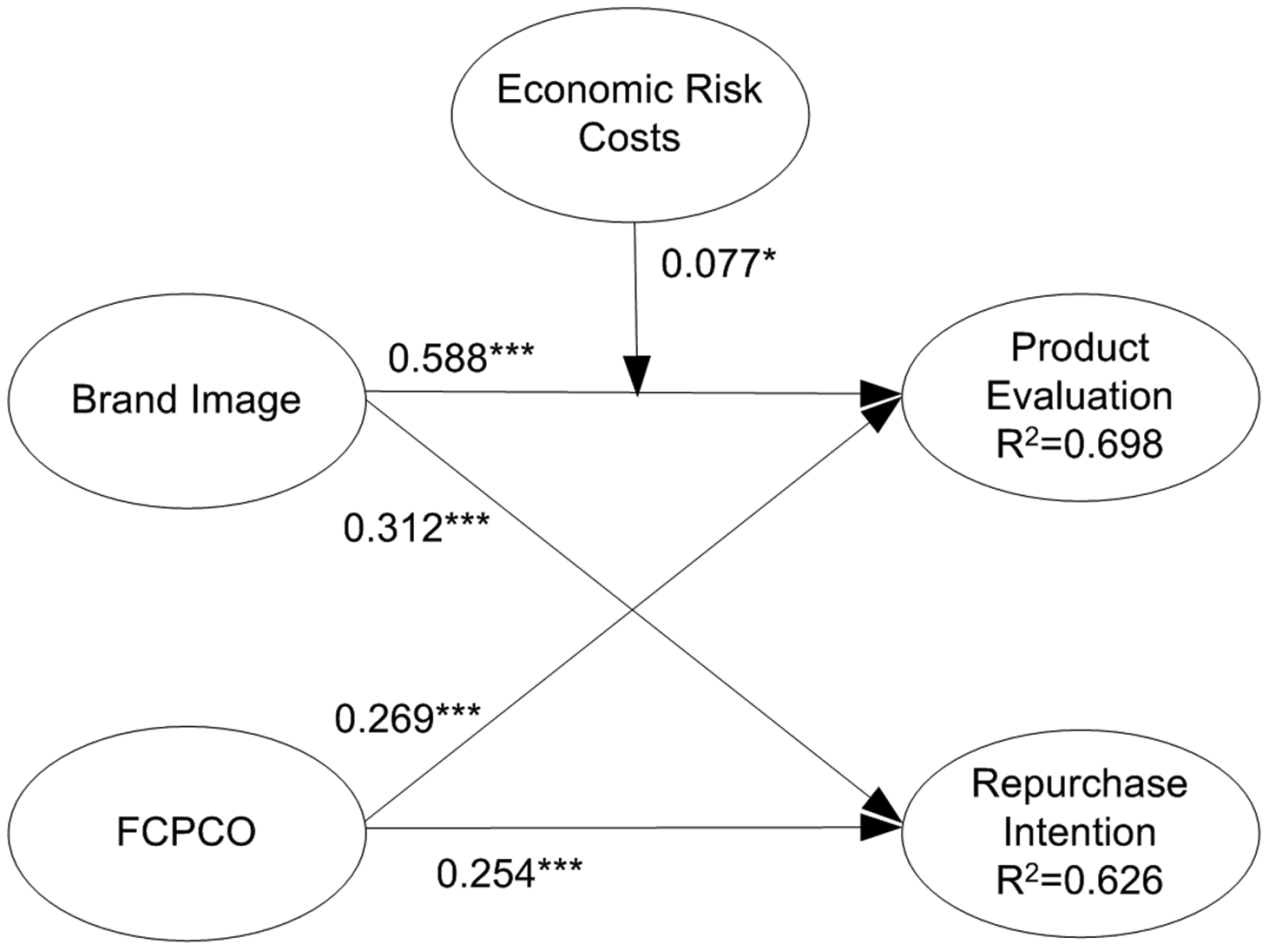
Economic risk costs’ moderating effect. Solid arrow is significant path; dotted arrow is nonsignificant path. ^*^p < 0.05; ^**^p < 0.01; and ^***^p < 0.001.

**Figure 6:**
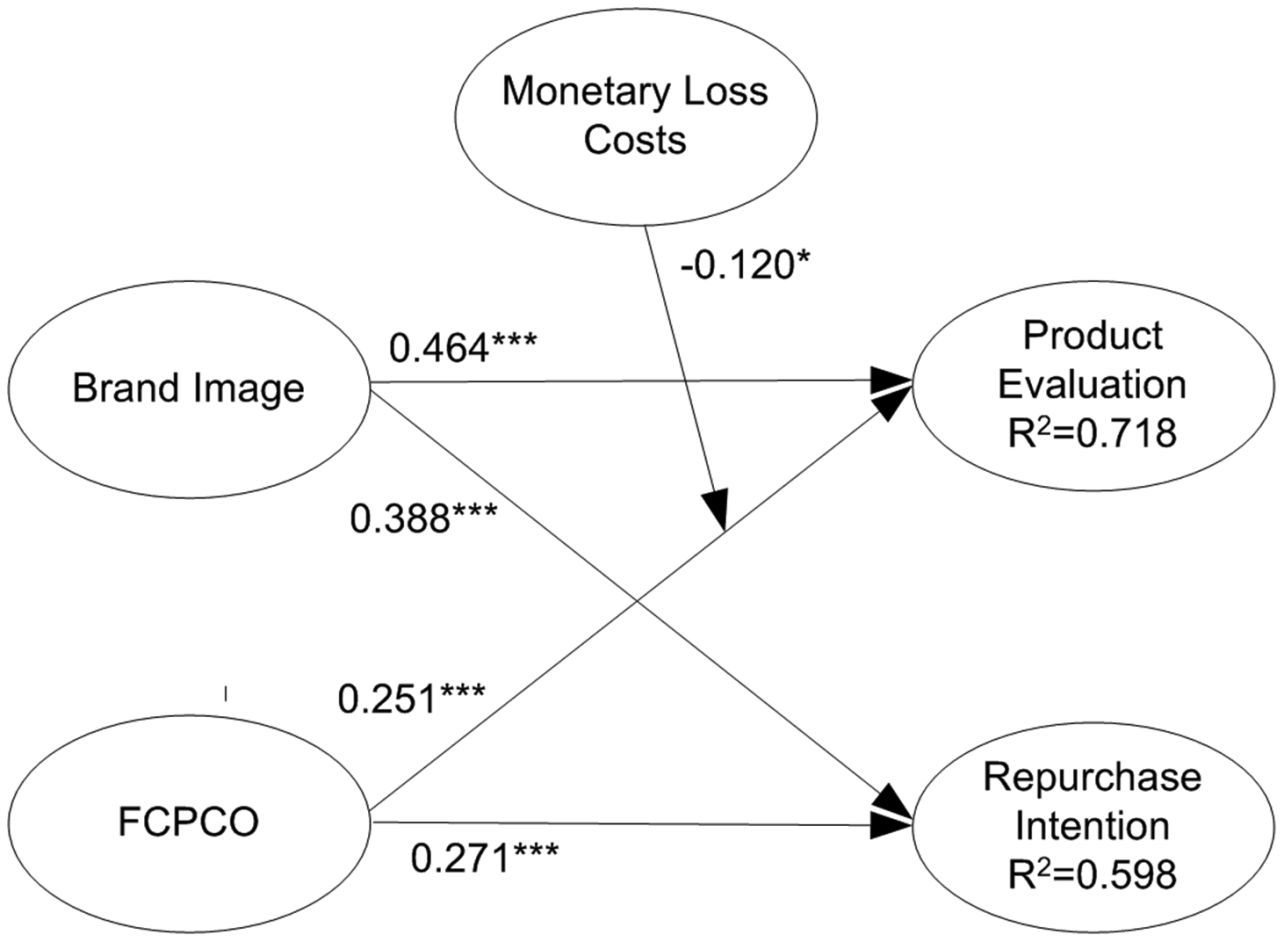
Monetary loss costs’ moderating effect. Solid arrow is significant path; dotted arrow is nonsignificant path. ^*^p < 0.05; ^**^p < 0.01; and ^***^p < 0.001.

**Figure 7:**
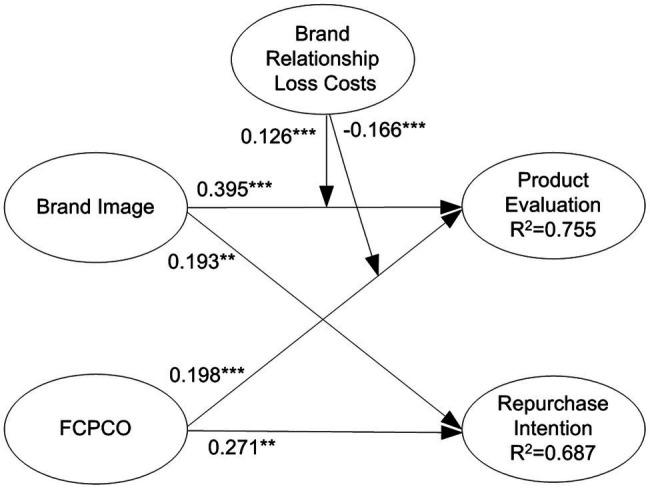
Brand relationship loss costs’ moderating effect. Solid arrow is significant path; dotted arrow is nonsignificant path. ^*^p < 0.05; ^**^p < 0.01; and ^***^p < 0.001.

By multiplying the moderating variables with corresponding indicators of predictor variables, interaction terms were created to test the moderating effects of switching costs. Subsequently, a model with moderating variables was compared with a model without moderating variables (Chin et al., 2003). The results reveal that only economic risk costs, monetary loss costs, and brand relationship loss costs had significant moderating effects. For clear visualization, only the statistically significant moderating effects are displayed in Figures 5–7. Economic risk costs exerted a positive moderating effect on the relationship between brand image and product evaluation ( β = 0.077, *p* < 0.05); monetary loss costs exerted a negative moderating effect on the relationship between FCPCO and product evaluation ( β = −0.120, *p* < 0.05); brand relationship loss costs exerted a positive moderating effect on the relationship between brand image and product evaluation ( β = 0.126, *p* < 0.001); and brand relationship loss costs exerted a negative moderating effect on the relationship between FCPCO and product evaluation ( β = −0.166, *p* < 0.001). Thus, H5-1, H5-3, H7-2, and H7-3 were partially supported.

## Discussion

This study developed and tested a novel theoretical framework for the moderating effects of switching costs. The developed framework provides international suppliers with new insights regarding marketing strategies. In particular, the findings of this study are of critical importance to South Korean corporations that have already entered or have plans to enter the Chinese market in that these findings can help them manage their relationship with customers. This study found that FCPCO and brand image positively affected product evaluation, which is in agreement with the finding of Koubaa et al. (2015) that brand image and information related to a product’s country of origin can positively affect product evaluation. The current study found that FCPCO and brand image positively affected repurchase intention, which is in agreement with the findings of previous studies that brand image can affect repurchase intention (Huang et al., 2019) and that country image can affect purchase intention (Bilkey and Nes, 1982; Roth and Romeo, 1992; Wang et al., 2012) and loyalty (Pappu et al., 2006). The present study compared the effects of brand image and FCPCO. On the basis of IPMA results, this study found that brand image had greater importance for product evaluation than FCPCO did, which is in agreement with the finding of some studies that brand image has a greater influence on product evaluation than does a product’s country of origin (Ulgado and Lee, 1993; Lee and Ganesh, 1999; Thakor and Lavack, 2003). However, the aforementioned finding of the current study contradicts the findings of some other studies, which have found that a product’s country of origin has greater importance for product quality evaluation than does brand image (Han and Terpstra, 1988; Tse and Gorn, 1993). Furthermore, the IPMA results of the present study indicated that FCPCO had a greater importance for repurchase intention than did brand image. A previous study also found that a product’s country of origin had a greater importance for repurchase intention than did price and other attributes (Okechuku and Onyemah, 1999). The IPMA results of the current study indicated that FCPCO had higher performance values than brand image did for product evaluation and repurchase intention. The findings of this study suggest that South Korean corporations that have already entered or plan to enter the Chinese market should prioritize brand image in their marketing strategies if they wish to achieve positive product evaluation by Chinese consumers. Alternatively, South Korean corporations should give priority to FCPCO in their marketing strategies if they desire to achieve high repurchase intention among Chinese consumers. Corporate executives can improve consumers’ familiarity with their brands through various approaches. For example, commercial advertisements are one method for improving brand recognition and sentiment and consequently sales. However, the effects of FCPCO on product evaluation and repurchase intention should not be ignored. At a national level, improving the overall demeanor of the population and deepening interactions with other countries are two methods for improving the image of a country’s citizens among foreigners (i.e., FCPCO), which in turn can increase the international acceptance of the country’s products. The results of this study on the moderating effects of switching costs were as follows: economic risk costs and brand relationship loss costs positively moderated the relationship between brand image and product evaluation; and monetary Loss costs and brand relationship loss costs negatively moderated the relationship between FCPCO and product evaluation. Other studies have also reported that procedural switching costs, financial switching costs, and relational switching costs moderate the relationships between satisfaction and repurchase intention and between customers’ perceived value and customer loyalty (Blut et al., 2015; El-Manstrly, 2016). The findings of this study on the moderating effects of switching costs have crucial theoretical and practical implications. South Korean corporations should develop marketing strategies that leverage the moderating effects of economic risk costs, monetary loss costs, and brand relationship loss costs to improve the relationship between brand image, FCPCO, and product evaluation in the Chinese market. In this study, the moderating effects of all types of switching costs except economic risk costs, monetary loss costs, and brand relationship loss costs were statistically nonsignificant. Thus, switching costs did not exert a moderating effect on repurchase intention outside the economic risk, monetary loss, and brand relationship loss dimensions. This result is consistent with those of some previous studies (Back and Lee, 2009; Ghazali et al., 2016). The aforementioned result may be attributed to the fact that repurchase was not considered an objective behavior in the current study. A study found that switching costs exhibited moderating effects only when repurchase was measured as an objective behavior. When repurchase was measured as repurchase intention, the moderating effects of switching costs were nonsignificant (Seiders et al., 2005). Moreover, the moderating effects of switching costs can be affected by situational variables, such as the types of trade, customers, and products; they are not always significant (Burnham et al., 2003; Yang and Peterson, 2004; Nie et al., 2018). An additional possibility is that in the commerce between South Korea and China, economic risk costs, monetary loss costs, and brand relationship loss costs are the dominant switching costs that exert moderating effects.

## Conclusion

This study makes the following academic and practical contributions to the fields of international commerce and marketing. First, to the best of our knowledge, the present study is the first to conduct in-depth research on FCPCO. This study expands the understanding on how FCPCO affects product evaluation and repurchase intention. Second, this study conducted a detailed examination into the multidimensional nature of switching costs and their moderating effects. In contrast to previous studies that mostly treated switching costs as a one-dimensional construct, the current study provides insights on the moderating effects of various types of switching costs. Finally, by delineating the moderating effects of switching costs on brand image, FCPCO, and product evaluation, the present study broadened the understanding of the moderating effects of switching costs, fills a gap in the literature.

This study has certain limitations that can be improved. First, the scope of this study was limited to South Korea’s commerce with China. Future studies should expand their scope to include other countries. Second, the current study was not based on a specific product or service type. Future studies are advised to investigate the moderating effects of switching costs in different industries, such as the cosmetic industry, mobile device industry, Internet service providing industry, banking industry, and hospitality industry. Third, this study relied on cross-sectional data. Future studies can consider using longitudinal data to conduct a more comprehensive analysis of how the moderating effects of switching costs vary with time. Fourth, the moderating effects of certain types of switching costs were found to be nonsignificant in the present study. Future studies can combine these switching costs with other factors to perform a more thorough investigation. Switching costs are complicated, and additional research is required on their moderating effects. Such research would provide additional insights regarding consumers’ switching costs, thereby facilitating sustainable corporate development.

## Data Availability Statement

The raw data supporting the conclusions of this article will be made available by the authors, without undue reservation.

## Ethics Statement

Ethical review and approval were not required for the study on human participants in accordance with the local legislation and institutional requirements. The patients/participants provided their written informed consent to participate in this study.

## Author Contributions

YS and RA conceived and designed the study. YS organized the database, performed the statistical analysis, and wrote the manuscript. RA helped with the references. All authors contributed to manuscript revision, read, and approved the submitted version.

## Conflict of Interest

The authors declare that the research was conducted in the absence of any commercial or financial relationships that could be construed as a potential conflict of interest.

## Publisher’s Note

All claims expressed in this article are solely those of the authors and do not necessarily represent those of their affiliated organizations, or those of the publisher, the editors and the reviewers. Any product that may be evaluated in this article, or claim that may be made by its manufacturer, is not guaranteed or endorsed by the publisher.
